# Power Output, Lactatemia, and Maximum Oxygen Consumption During a Specific Off-Water Incremental Test in International-Level Podium-Winner Kayak and Rowing Athletes

**DOI:** 10.3390/jfmk10020203

**Published:** 2025-06-01

**Authors:** Oscar Crisafulli, Matteo Fortunati, Tiziano Gemelli, Luca Grattarola, Venere Quintiero, Massimiliano Febbi, Patrik Drid, Stefano Ramat, Giuseppe D’Antona

**Affiliations:** 1 Centro di Ricerca Interdipartimentale Attività Motorie e Sportive (CRIAMS)-Sport Medicine Centre Voghera, University of Pavia, 27058 Voghera, Italy; oscar.crisafulli@unipv.it (O.C.); matteo.fortunati01@gmail.com (M.F.); tiziano.gemelli@unipv.it (T.G.); lucagrattarola.96@gmail.com (L.G.); venere.quintiero01@universitadipavia.it (V.Q.); stefano.ramat@unipv.it (S.R.); 2 Faculty of Sport and Physical Education, University of Novi Sad, 21000 Novi Sad, Serbia; patrikdrid@gmail.com; 3 Department of Industrial Engineering, University of Tor Vergata, 00133 Rome, Italy; massimilianofebbi@gmail.com; 4 Laboratory for Rehabilitation, Medicine and Sport (LARM), 00133 Rome, Italy; 5 Department of Electrical, Computer and Biomedical Engineering, University of Pavia, 27100 Pavia, Italy; 6 Department of Public Health, Experimental and Forensic Medicine, University of Pavia, 27100 Pavia, Italy

**Keywords:** cardiopulmonary evaluation, high-performance athletes, incremental exercise test, boat sport, maximal oxygen consumption

## Abstract

Background: To achieve victory, kayaking and rowing athletes must develop optimal aerobic conditioning and the capacity to sustain anaerobic work production. To assess these characteristics, power output (PO), lactatemia response, and maximum oxygen uptake (VO_2_max) are usually measured. The goal of this research is to report the values of PO, lactatemia, and VO_2_max—expressed in relative, absolute, and body size-scaled values—in successful international-level athletes to provide reference values for those striving to compete at the highest level. Methods: A total of 15 international-level medallist boat sports athletes were recruited: 8 male kayakers (age 21 ± 3 years, height 181.7 ± 5.3 cm, body mass 78.7 ± 5.6 kg), 2 female kayakers (age 22 ± 2 years, height 168.0 ± 2.8 cm, body mass 64.9 ± 2.7 kg), and 5 male rowers (age 20 ± 1 years, height 181.9 ± 4.7 cm, body mass 83.9 ± 7.3 kg). The athletes’ PO, lactatemia, and VO_2_max were assessed using an off-water, sport-specific cardiopulmonary test on a paddle and rowing ergometer. Results: Respectively, in male and female kayakers and male rowers, maximum lactatemia was 11.9 ± 2.2 mmol/L, 9.3 ± 3.6 mmol/L, and 13.2 ± 3.7 mmol/L; maximum PO was 225.0 ± 13.4 W, 162.5 ± 31.8 W and 432.0 ± 33.5 W; and VO_2_max was 57.6 ± 5.4 mL/min/kg, 52.2 ± 1.0 mL/min/kg, and 63.7 ± 11.7 mL/min/kg. VO_2_max scaled by body size was, respectively, 311 ± 39 mL/kg^0.67^/min, 319 ± 15 mL/kg^0.67^/min, and 330 ± 72 mL/kg^0.67^/min. Conclusions: This study is the first to report the values of PO, lactatemia, and VO_2_max—expressed in relative, absolute, and body size-scaled values—assessed during a sport-specific cardiopulmonary test in international-level boat sports athletes. These values could be a preliminary reference guideline for optimal cardiorespiratory conditioning in athletes aiming at international-level competitions.

## 1. Introduction

Canoe and kayak competitions are divided into several disciplines, each with unique characteristics and challenges. The two main categories are canoe sprint and canoe slalom. Canoe sprint takes place on flatwater and involves racing over set distances in straight lines. Athletes compete in either canoes or kayaks in single, double, or four-person boats [1]. Canoe slalom, on the other hand, is held on whitewater courses where athletes navigate through a series of hanging gates, aiming for speed and precision while avoiding time penalties [2]. Olympic flat-water kayak (K) races over two distances, K500 m and K1000 m. The current world records (WRs) for the men’s K500 and K1000 are 1:35.04 s and 3:20.643 s, respectively, while, for the women, the WRs stand at 1:46.19 s for the K500 and 3:48.560 s for the K1000 [3]. The energetic contribution was shown to be 30–40% anaerobic during the female K500 m [4], and 15% in the male K1000 m [5]. This shows that the longer the distance, the lower the anaerobic contribution [6].

Rowing competitions are water sports where athletes propel boats using oars, competing over set distances—most commonly 2000 m in Olympic events. The sport is broadly divided into two main types: sweep rowing and sculling. In sweep rowing, each rower holds one oar with both hands and rows on one side of the boat. This includes boat classes like the pair, four, and eight. In sculling, each athlete uses two oars, one in each hand, with boat classes including the single scull, double scull, and quadruple scull. Competitions are held on straight, calm-water courses, and races are won by the first boat to cross the finish line [7]. Moreover, rowing competitions are categorised based on different body sizes, termed lightweight (maximum individual body masses of 72.5 kg), open, or heavyweight (with no restriction on body mass) [7]. Rowing race WRs in the men’s and women’s single scull were, respectively, 6:30.74 s and 7:07.71 s [8]. The anaerobic energetic contribution of this sport is around 13% in male oarsmen [9], while, to the authors’ knowledge, no study has been conducted on cohorts of female rowers. This contribution reflects a prevalent aerobic metabolism [10]. Therefore, competitive international-level athletes in kayaking and rowing must engage in daily and extremely intense workouts to develop optimal cardiorespiratory conditioning [5,7,9,11,12].

Maximal oxygen consumption (VO_2_max) is the capacity of the circulatory, respiratory, and muscular systems to deliver and utilise oxygenated blood to the working tissues and muscles during an activity [13,14]. VO_2_max is primarily defined as the maximal oxygen uptake reached during incremental exercise, characterised by a plateau in oxygen consumption maintained for at least 30 s despite increasing workload [15]. If these criteria are not met, the following secondary criteria must be achieved: heart rate (HR) within 10 beats/min or 5% of the age-predicted (220-age) HR maximum, lactatemia concentration above 8 mmol, and the respiratory exchange ratio (RER) above 1.0 [15]. VO_2_max is commonly expressed linearly based on body mass. Thus, absolute values (mL/min) are divided by body mass raised to the power of 1 (ml/kg/min). This classical relative calculation of VO_2_max has been extensively utilised in various contexts, including clinical populations [16,17,18], and normative reference values have been established for healthy individuals [19,20] and athletes [21]. However, research suggests that VO_2_ increases in proportion to body mass raised to a power between 0.66 and 0.75, following a surface area-to-volume ratio, rather than in proportion to body mass raised to 1 [22,23,24] because humans have a highly irregular shape (i.e., with various lengths and widths being not uniform throughout the structure) [25]. Therefore, it is suggested to divide the absolute value of VO_2_max by body mass elevated with an exponent of 0.67 when the subjects being compared are of similar height, age, and training background, and an exponent of 0.75 for more heterogeneous groups [23]. This calculation allows for removing body size as a variable influencing the aerobic capacity value [25] as shown in a large cohort of endurance athletes [26]. For example, considering two athletes with different body masses (100 kg and 60 kg), but with identical VO_2_max expressed linearly 45 mL/kg/min obtained from a VO_2_max of, respectively, 4.5 L/min and 2.7 L/min, it shows an allometric value of 205.7 mL/kg^0.67^/min and 173 mL/kg^0.67^/min, thus indicating a better cardiorespiratory fitness in the heavier athlete, despite an identical value if expressed linearly [25]. Therefore, its scaled calculation is requested to compare cardiorespiratory fitness between athletes of different body masses [25]. In practice, this calculation allows the trainer to avoid underestimating the VO_2_max of heavier athletes compared to lighter subjects, which could possibly ameliorate training prescription [25]. This might emerge as particularly relevant within boat sport athletes, such as rowers. Indeed, different body sizes among athletes are common (i.e., lightweight and heavyweight) [7]. Additionally, VO_2_max scaled by body size can be a guideline that indicates good conditioning in sports athletes [27]. Despite this valuable information, scaled VO_2_max by body size has been only used in athletic populations [28,29,30,31], and no studies, to the authors’ knowledge, have been conducted in sedentary or clinical populations. As a consequence, the widespread knowledge of this scaled parameter may be limited.

VO_2_max is correlated with performance in sports with a high aerobic contribution [32], and, specifically, it is associated with racing time in sprint kayaking [4] and rowing speed in 2000 m [33]. Consequently, this physiological parameter is believed to be a determining factor in achieving victory. Therefore, the VO_2_max has already been described in several cohorts of well-trained [34], club level [35], and international-level kayakers [4,5,11,35,36,37,38,39,40,41], along with novice and collegiate rowers [42,43], and élite oarsmen [9,12,44,45], showing very high values compared to a large cohort of healthy subjects [46] and similar to other sports team athletes [47]. However, no studies have scaled VO_2_max by body size in a cohort of international-level kayakers and rowers, leaving a reference value for optimal cardiorespiratory fitness in these élite-level cohorts of athletes unknown.

Racing performance in boat sports also depends on the athlete’s capacity to sustain anaerobic work production, especially in shorter races [4]. To assess this capacity, the response of lactatemia at different power outputs (POs) has been commonly evaluated in athletes as it is strictly correlated with performance in endurance sports [48]. Particularly, in boat sport competitors, lactatemia at specific thresholds and maximum PO (POmax) is usually reported [4,37,45,49,50,51,52,53]. In this context, the intensity at which there is a sustained and exponential increase in blood lactate concentration—indicating a shift toward predominantly anaerobic metabolism—is referred to as the second lactate threshold (LT_2_) [48]. Notably, PO at LT_2_ is correlated with sprint performance in well-trained kayakers [34]. Additionally, lactatemia concentration at a fixed value of PO decreases with training, as shown in several studies [49,54,55]; therefore, it is expected that élite-level athletes show superior values of PO, at the same amount of lactatemia response, compared to non-élite counterparts. In this regard, elite male kayak paddlers were compared to strength athletes body builders, and physically active healthy subjects on an arm crank ergometer. Results reported that the kayakers exhibited a significantly lower lactatemia concentration at all POs [56], suggesting that either the training regime (endurance athlete vs. power athlete) or the physical conditioning (trained vs. less trained) could play an important role in lactatemia concentration.

Consequently, providing reference physiological response data for trainers evaluating physical conditioning through a ramp test for kayak and rowing athletes would be valuable for a direct comparison. Therefore, the aim of this observational research is to report benchmark values of PO, lactatemia, VO_2_max—expressed in relative, absolute, and body size-scaled values—measured during a cardiopulmonary incremental test in international-level podium-winner boat sport athletes.

## 2. Materials and Methods

### 2.1. Participants

A total of 15 boat sports athletes were recruited in this study. Specifically, they were grouped into three subgroups as follows: 8 male kayakers (mean ± DS (range), age 21 ± 4 (18–30) years, height 181.7 ± 5.3 (177.0–191.0) cm, body mass 78.7 ± 5.6 (72.0–88.0) kg); 2 female kayakers (age 22 ± 2 (21–24) years, height 168.0 ± 2.8 (166–170) cm, body mass 64.9 ± 2.7 (63.0–66.8) kg); and 5 male rowers (age 20 ± 1 (19–22) years, height 181.9 ± 4.7 (177.5–189.6) cm, body mass 83.9 ± 7.3 (73.8–93.0) kg). All subjects were international-level athletes of kayak and rowing who participated in the 2020 European Championship (October 2020, Poznan) and held at least one podium win in major international competitions (world specialty challenges). Overall, during their careers, the kayakers achieved a total of 15 medals (5 gold, 5 silver, and 5 bronze). While the oarsmen cumulatively won 6 medals (2 silver and 4 bronze). The inclusion criteria were being free from physical limitations, health problems, or musculoskeletal injuries that could negatively interfere with regular performance. The measurements were conducted at the CRIAMS sport medicine centre, University of Pavia, Italy. Participants were required to abstain from alcohol and unaccustomed exercise for 48 h before all experimental trials. The procedures were conducted in July 2021, in the middle of the competition phase of the annual periodisation plan of each athlete’s season, which would lead to the period of peak performance for the upcoming national and international appointments. All volunteers read and signed an informed consent form approved by the ethics committee. The research protocol was approved by the Sport and Physical Education Ethics Committee of the University of Novi Sad, Serbia (No. 47-06-02/2021-1; 22 June 2021) in compliance with the ethics standards committee. Data and sensitive information were protected according to the General Data Protecting Regulation (GDPR).

### 2.2. Physiological Assessment

#### 2.2.1. Cardiopulmonary Exhaustion Test on Paddle Ergometer

The kayak athletes (males, *n* = 8; females, *n* = 2) performed an off-water, cardiopulmonary exhaustion test on a calibrated paddle ergometer (Dansprint ApS, Dansprint^®^, Hvidovre, Denmark). Before each test, the position of the ergometer’s foot bar was adjusted to resemble the kayak used in the water. After 5 min of warm-up paddling at an intensity of 60 W for males and 40 W for females, the first stage of the incremental test started at a PO of 75 W for males with increments of 25 W until exhaustion, while females were given an initial PO of 50 W with steps of 15 W. Each stage lasted 2 min, and it was conceived to provoke exhaustion within 6–8 stages. The external load was set in accordance with previous research on male and female kayakers [4,35,57] and with extensive field observation of the athletes’ training.

#### 2.2.2. Cardiopulmonary Exhaustion Test on Rowing Ergometer

The group of oarsmen (males, *n* = 5) performed an off-water, cardiopulmonary exhaustion test on a calibrated rowing ergometer (Indoor Rower Concept2, Concept2^®^, Morrisville, VT, USA). After 5 min of warm-up rowing at an intensity of 160 W, the first stage of the incremental test started at a PO of 200 W with increments of 40 W until exhaustion. Each stage lasted 2 min and was conceived to provoke exhaustion within 6–8 stages. The external load was set in accordance with a previous study on rowers [12] and with extensive field observation of the athletes’ training.

It was impossible to set a fixed resistance in the paddle and the rowing ergometer; therefore, athletes had to adapt their PO by looking at the display before them, keeping it in an acceptable range during each stage. Athletes were encouraged to complete as many stages as possible. Specifically, the test was considered maximal when it met the following three criteria: respiratory exchange ratio (RER) > 1.1, ratio of perceived exertion (RPE) ≥ 8, and VO_2_ at plateau for at least 30 s. Notably, RER was derived from raw values, while VO_2_max was calculated as the average of the 30 s following achievement of RER = 1.1 and RPE ≥ 8 [15,58,59]. The extrapolated data refer to the last increment completely performed. The cool-down procedure included 15 min of total rest after exhaustion. The absolute value of VO_2_max (mL/min) was then divided, respectively, by body mass to obtain the classical relative calculation (mL/kg/min) and by body mass elevated by an exponent of 0.67 (mL/kg^0.67^/min) to obtain the body size-scaled measure. The exponent of 0.67 was utilised because the athletes were of similar height, age, and training background [25]. All testing sessions were performed at the same time of the day (from 09:30 to 12:30 am). Each test session was conducted under standard environmental conditions (18–22 °C; 760 mmHg; 40–45% atmospheric humidity).

Pulmonary gas exchange. Respiratory gas exchange parameters (O_2_ uptake (VO_2_), carbon dioxide production (VCO_2_), and minute ventilation (VE) were continuously recorded breath by breath using the Cosmed Quark B2 mask (Cosmed^®^, Roma, Italy). Beat-to-beat heart rate was monitored throughout the test by ECG. The data were then analysed and extrapolated using the Cosmed Omnia software (ver. 1.6.10—Cosmed^®^, Roma, Italy). The second ventilatory threshold (VT_2_) was identified by an increase in the ventilatory equivalent of oxygen (VE/VO_2_) and carbon dioxide (VE/VCO_2_) combined with a reduction in the end-expiratory pressure of carbon dioxide (PETCO_2_) [60].

Lactatemia concentration. Lactatemia concentration was measured using the Lactate Pro 2 instrument (Arkray^®^, Kyoto, Japan). It is a handheld point-of-care analyser that operates by enzymatic amperometric detection. Lactatemia reacts with the reagent on the test strip (Arkray Lactate Pro 2 Test Strip, Arkray^®^, Kyoto, Japan), which produces a small electrical current proportional to the concentration of lactatemia. The metre measures this current and calculates the lactatemia level. It requires 0.3 μL of a whole-blood sample and 15 s to measure the lactatemia value. The lactate Pro 2 instrument has a measurement range between 0.5 and 25.0 mmol/L. Capillary lactate measurement at the earlobe obtained from this device has good agreement with a reference method in healthy volunteers [61,62], along with a within-sample device coefficient of variation less than 1.0% [63] Lactatemia analysis was conducted at rest (basal assessment), immediately after the 5 min fixed warm-up phase, and immediately after each stage. The start of the incremental test coincides with the end of the warm-up phase plus the time requested to collect lactatemia (approximately 8–12 s). During transitions from the warm-up to the first stage, and between each subsequent test stage, athletes paused exercise only for the time needed to perform the lactatemia collection before resuming the prescribed workload.

Lactate threshold identification. LT_2_. The D_MAX_ method was used to calculate LT_2_. This method was determined by plotting the lactatemia response (mmol/L) to exercise intensity (PO) in a third-order polynomial regression curve. The D_MAX_ was defined as the point on the regression curve that yields the maximal distance to the straight line formed by the two end points of the curve [64,65]. This concept is highly repeatable (intra-subjects’ coefficient of variation of 3.4%, Cronbach’s alpha 0.96) [65].

### 2.3. Statistical Analysis

According to the Shapiro–Wilk test, all variables were not normally distributed; therefore, the data are summarised by median (25–75th interquartile range) and 95% confidence intervals. The VO_2_max scaling by body size was calculated in previous studies by multiplying the average VO_2_max by 100 and dividing the result by the mean body mass raised to an exponential of 0.67. For example, a study that reported a mean body mass of 88.9 kg and a VO_2_max of 66.3 mL·Kg·min^−1^ [44], the calculation was as follows:

$$\frac{\left(66.3\;\times \;100\right)}{88.9^{0.67}}$$

All the analyses were performed using the Jamovi software (version 2.3) (The Jamovy Project, Newcastle, New South Wales, Australia) [66] and Microsoft Excel^®^ (Microsoft, Redmond, WA, USA).

## 3. Results

Performance/physiological responses assessed during the off-water paddle ergometer and rowing ergometer cardiopulmonary exercise testing, reported from each subgroup of athletes, with the calculation of VO_2_max scaled by body size, are shown in Table 1.

## 4. Discussion

The study presented here is the first to quantitatively calculate VO_2_max scaled by body size in an international-level cohort of kayak and rowing athletes obtained during a sport-specific off-water cardiopulmonary exercise test. The main finding is that our cohort shows very high values of VO_2_max—expressed in relative, absolute, and body size-scaled values—in line with previously reported values [4,35,36,37,44,45], indicating excellent cardiorespiratory conditioning. Moreover, PO and lactatemia at LT_2_ and maximum intensity are reported.

### 4.1. Maximal Oxygen Consumption

The subgroup of male kayakers obtained a VO_2_max in line with that cited in a recent review [11]; however, it was inferior to the value measured in a small sample representing some of the best under-23 athletes competing for France [5]. The difference found between the two studies could be related to the protocol adopted by Zouhal et al. (2012), in which kayakers were assessed during incremental on-water paddle testing [5]. In fact, it is known that athletes perform better in real conditions. Thus, results derived from a non-specific evaluation may lose some validity. Referring to the female subgroup, the results show a VO_2_max similar to that found in several investigations on elite-level female kayakers [4,36,37]. Also, the subgroup of rowing athletes has a VO_2_max matched with that measured in a cohort of international-level competitors; however, the value reported by Lindenthaler et al. (2018) depicted a reduced standard deviation compared to our results, which could be due to a greater homogeneity in terms of cardiopulmonary adaptations [44]. However, it must be considered that our sample size was smaller (*n* = 5) than that of the comparator study, which included 20 oarsmen [44]. This may have led to greater dispersion (i.e., higher standard deviation) in the results. Finally, within our rowers, an athlete reached a value of 79.2 mL·kg^−1^·min^−1^, which reflects very high conditioning seen in endurance athletes such as cross-country skiers [67] or triathlon [68]. Summing up the results, our participants possess an aerobic capacity comparable to that of international-level boat sports competitors; thus, these values could be taken as suggestive of proper cardiorespiratory conditioning in a similar cohort of athletes.

Importantly, considering the VO_2_max scaled by body size, our results are similar to those calculated by us in other studies about males [5,35,57] and female kayakers [4,37], and rowers [9,12,44,45]. Therefore, the VO_2_max scaled by body size proposed in this research could be taken as an initial reference for those aspiring to compete in international races.

### 4.2. Second Ventilatory Threshold

Comparing VO_2_VT_2_ with boat sports literature, it seems optimised in female kayakers based on the fact that it was detected at approximately the same percentage of VO_2_max in a small group of international-level female paddlers [4]. On the contrary, based on the same criteria, our male kayakers and oarsmen show VO_2_VT_2_ at an inferior relative intensity of cardiorespiratory fitness than, respectively, an equivalent cohort of paddlers [35], and rowers [38,45], suggesting that it can be further ameliorated.

### 4.3. Power Output and Lactatemia

During the specific, off-water cardiopulmonary exercise test, the subgroup of male kayakers achieved a POVT_2_ similar to that reported in the literature [35], but exhibited lower maximum lactatemia compared to a small cohort of age-matched kayakers undergoing an open water incremental test [5]. However, methodological differences were observed with the latter study, such as the intensity being prescribed based on boat velocity. Female kayakers demonstrated values of POmax and maximum lactatemia comparable to those reported in the literature in a similar off-water [4,37] and on-water [36] incremental test. Finally, the subgroup of oarsmen achieved POmax and maximum lactatemia slightly lower than a similar cohort [44]; however, they underwent discontinuous incremental exercise tests, introducing methodological differences in the protocol and variable assessments. Nonetheless, compared to a similar cardiopulmonary exercise test, the results are consistent [45]. Notably, our LT_2_, based on the D_MAX_ method, was reached in all subgroups at a percentage of POmax (<75%), largely inferior compared to those of world-class swimmers [69]. Indeed, in these exceptional elite athletes, it was reported that LT_2_ was reached at around 97% of their maximum swimming speed [69], thus suggesting a higher level of conditioning should be reached by elite athletes.

### 4.4. Limitations and Future Directions

We acknowledge that this research has some limitations. For instance, the reduced sample size of participants, as well as their unique physical characteristics, did not allow the results to be generalised to boat sports cohorts of different levels of performance. Further descriptive studies must be conducted in a larger cohort of boat sport athletes to increase benchmark reference values. Another limitation could be the fact that the paddle and rowing ergometer used for this investigation does not allow setting a fixed resistance (i.e., PO) in each stage, but the athletes, as written in the dedicated section, had to adapt their PO by looking at the display before them, keeping it within an acceptable range. Therefore, future studies should utilise an instrumentation in which PO could be fixed at each stage.

## 5. Conclusions

This is the first study that reports the values of VO_2_max—expressed in relative, absolute, and body size-scaled values—measured during a specific, off-water cardiopulmonary incremental test in a homogeneous cohort of medallist international-level kayak and rowing athletes. Moreover, PO and lactatemia at LT_2_ and at maximum intensity are reported. Although these data were obtained from a small group, they reflect the physical conditioning of athletes who have competed and won a podium in world specialty challenges. Consequently, athletes in boat sports aspiring to compete internationally are encouraged to aim for the values outlined in this research.

## Figures and Tables

**Table 1:** Performance/physiological responses of international-level kayak and rowing athletes.

Variables	Kayak		Rowing
	Males (*n* = 8)	Females (*n* = 2)	Males (*n* = 5)
VO_2_max (L/min)	4.45 (4.30–4.75) [4.30, 4.90]	3.35 (3.32–3.38)	5.10 (5.10–5.40) [4.3, 6.6]
VO_2_max (mL/kg/min)	58.8 (52.6–61.0) [51.70, 61.80]	52.2 (51.9–52.5)	58.0 (55.1–73.0) [53.10, 79.20]
VO_2_max (mL/kg^0.67^/min)	324 (277–333) [267.74, 343.03]	319 (314–324)	288 (280–409) [264.40, 409.03]
VO_2_VT_2_ (mL/min)	3045 (2820–3385) [2764.00, 3431.00]	3001 (2973–3029)	3723 (3392–3780) [3047.00, 4361.00]
VO_2_VT_2_ (%VO_2_max)	69.4 (64.9–71.9) [56.45, 72.53]	88.6 (88.4–88.8)	72.6 (62.9–85.1) [46.16, 87.99]
POmax (W)	225 (225–225) [225.00, 225.00]	163 (151–174)	440 (400–440) [400.00, 480.00]
POVT_2_ (W)	150 (144–175) [125.00, 175.00]	125 (118–133)	320 (240–320) [240.00, 360.00]
POVT_2_ (%POmax)	68.3 (63.9–75.7) [55.56, 77.78]	77.1 (76.4–77.8)	72.7 (60.0–80.0) [50.00, 81.82]
POLT_2_ (W)	151 (142–157) [135.80, 159.90]	113 (109–116)	314 (305–320) [299.70, 353.70]
POLT_2_ (%POmax)	66.2 (60.1–69.6) [59.48, 71.07]	70.1 (67.4–72.7)	73.7 (69.2–78.5) [68.11, 80.05]
Lactatemiamax (mmol/L)	12.0 (10.6–12.7) [10.20, 13.10]	9.35 (8.07–10.6)	12.3 (12.1–14.6) [8.40, 18.40]
LactatemiaLT_2_ (mmol/L)	2.92 (2.61–3.34) [2.44, 3.37]	2.47 (2.32–2.62)	3.92 (2.66–3.99) [2.59, 4.04]

Data are represented as median (25th–75th interquartile range) [95% confidence interval]. L/min litre/minute; mL/min/kg; millilitre/minute/kilogram; % percentage; Lactatemiamax lactatemia maximum; LactatemiaLT_2_ Lactatemia at second lactate threshold (calculated with D_MAX_ method); mmol/L millimole/litre; W watt; POVT_2_ power output at second ventilatory threshold; POmax maximum power output; POLT_2_ power output at second lactate threshold (calculated with D_MAX_ method); VO_2_max maximal volume of oxygen consumed either as absolute value, and in relative values with body mass elevated to the power of 1 (mL/kg/min) and to a power of 0.67 (mL/kg^0.67^/min); VO_2_VT_2_ volume of oxygen consumed at second ventilatory threshold.

## Data Availability

The data associated with the paper are not publicly available but are available from the corresponding author (G.D.A.) upon reasonable request.
